# Supplementary figures and images for: GWAS and 3D chromatin mapping identifies multicancer risk genes associated with hormone-dependent cancers
Source: PLoS Genet. 2024 Nov 25;20(11):e1011490. doi: 10.1371/journal.pgen.1011490 (PMC11627375; doi:10.1371/journal.pgen.1011490)

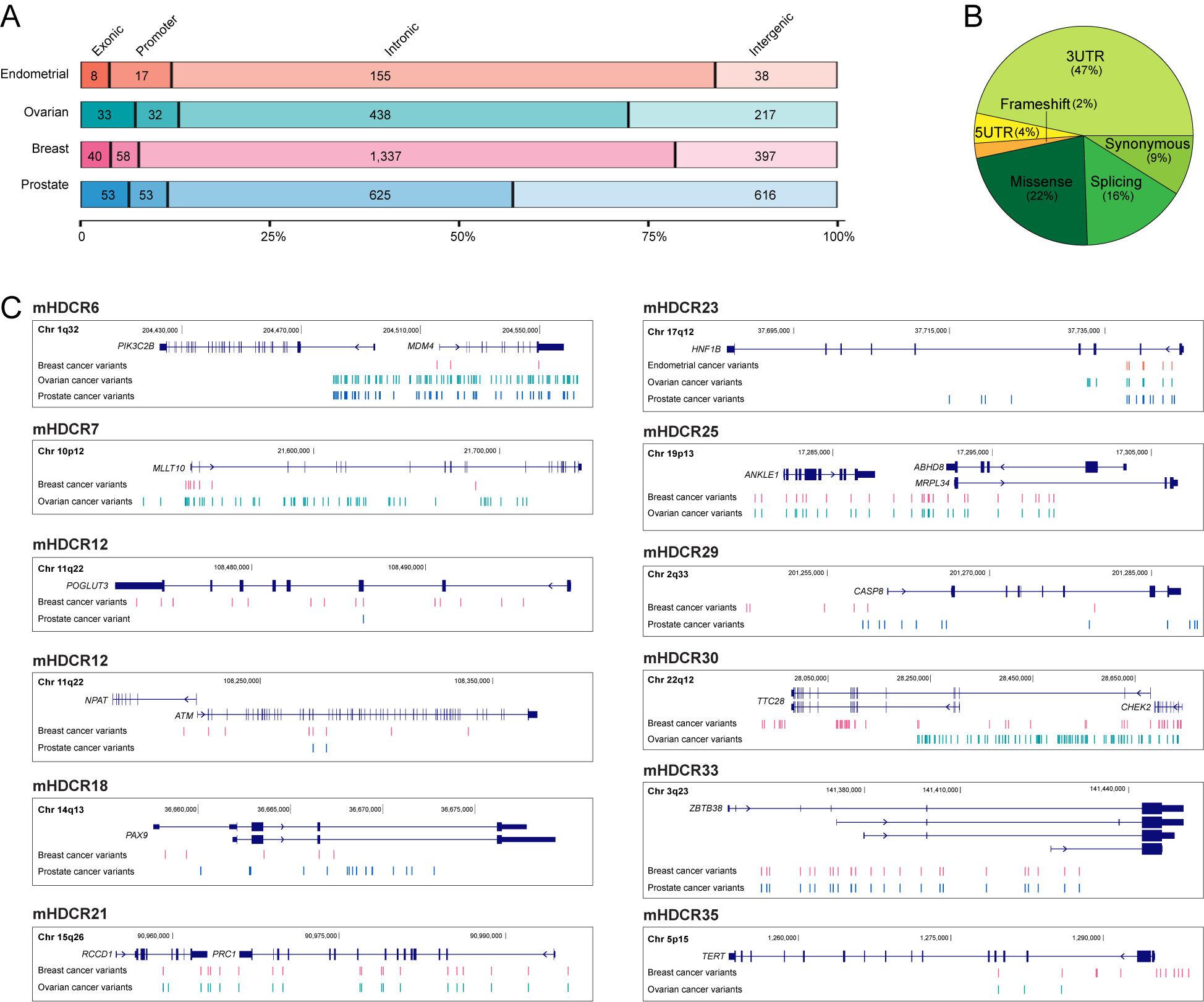

Supplement: S1 Fig — (A). The numbers and percentage of risk variants located in exons, promoters (TSS ± 2 kb), introns and intergenic regions. (B). The percentage of exonic risk variants in different functional categories. (C). Schematic representation of the 17 candidate mHDCR genes that contain exonic, promoter or potential splicing variants associated with two or more HDCs. WashU genome browser (hg38) showing GENCODE annotated genes (blue) and risk variants as coloured vertical lines (pink-breast cancer; orange-endometrial cancer; teal-ovarian cancer; blue-prostate cancer). (TIF) [file pgen.1011490.s001.tif]

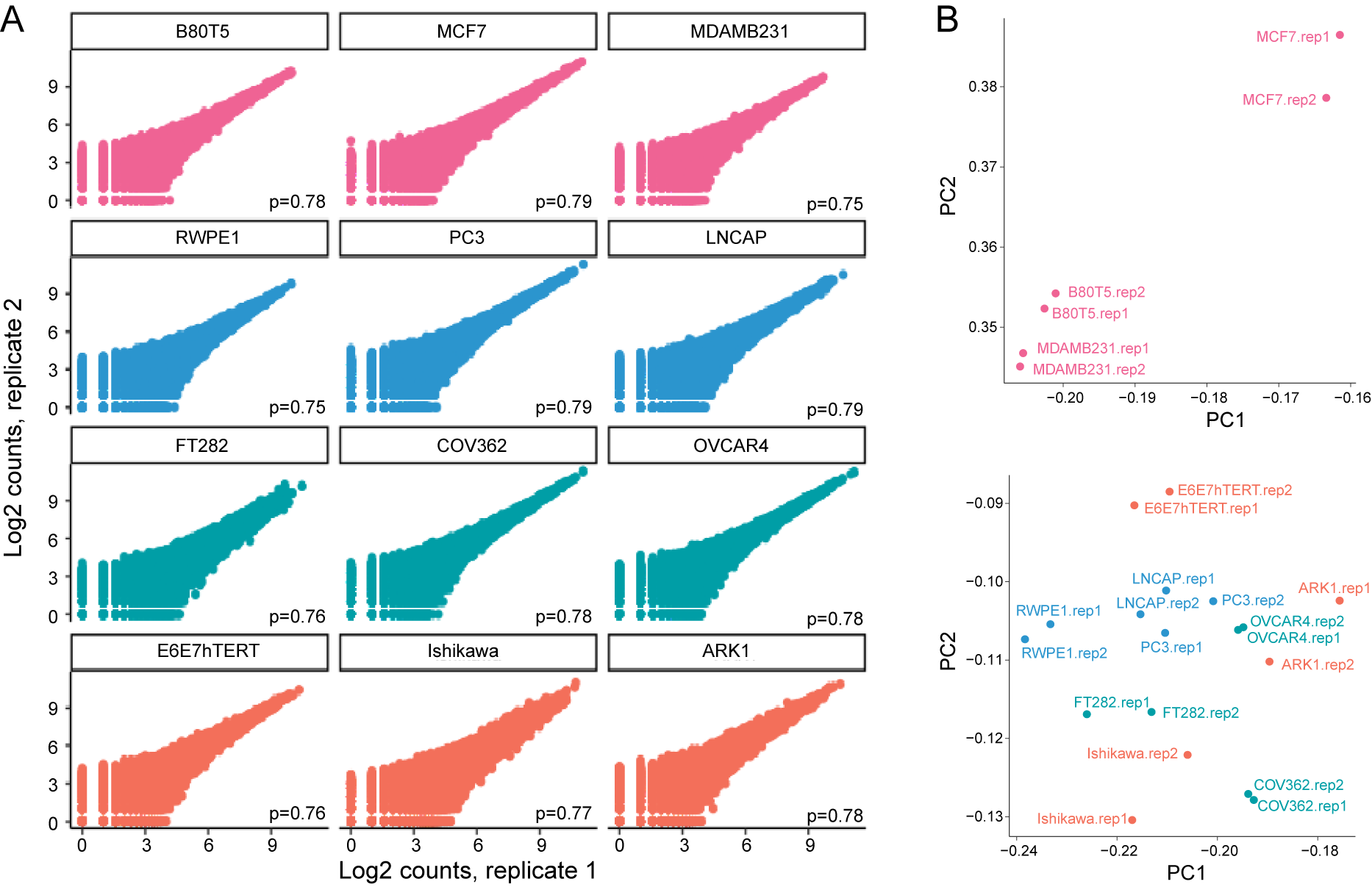

Supplement: S2 Fig — (A). Scatter plots showing the correlation between duplicate PCHiC libraries based on the number of raw di-tags mapping to interaction fragment pairs. ρ is Spearman’s correlation. (B). Principal component analysis (PCA) of CHiCAGO-scored interactions in PCHiC biological replicates for breast cells lines (top panel) or prostate, ovarian and endometrial cell lines (bottom panel). (TIF) [file pgen.1011490.s002.tif]

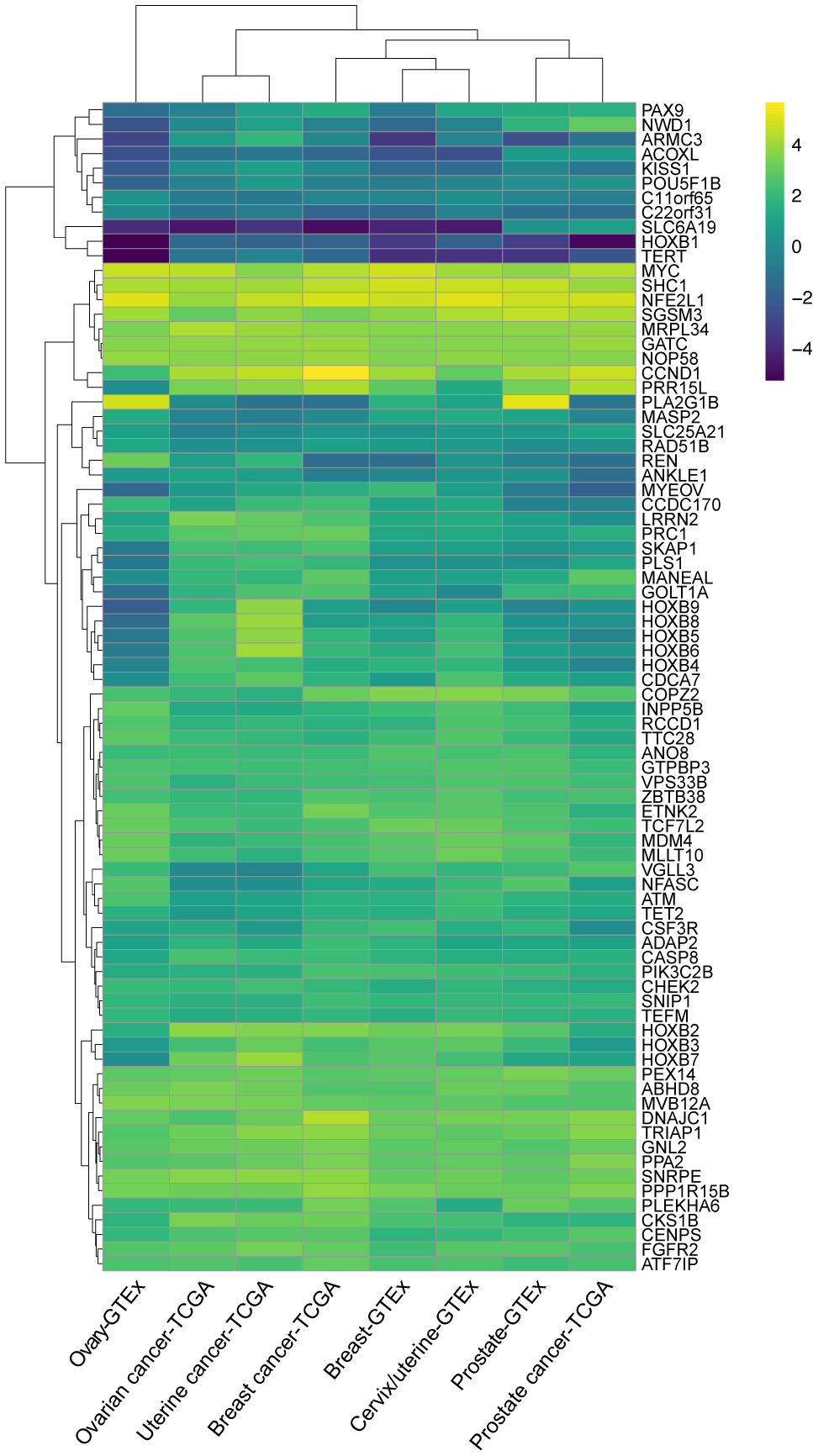

Supplement: S3 Fig — Heatmap showing candidate mHDCR gene expression from normal tissue samples in GTEx and tumor samples in TCGA. (TIF) [file pgen.1011490.s003.tif]

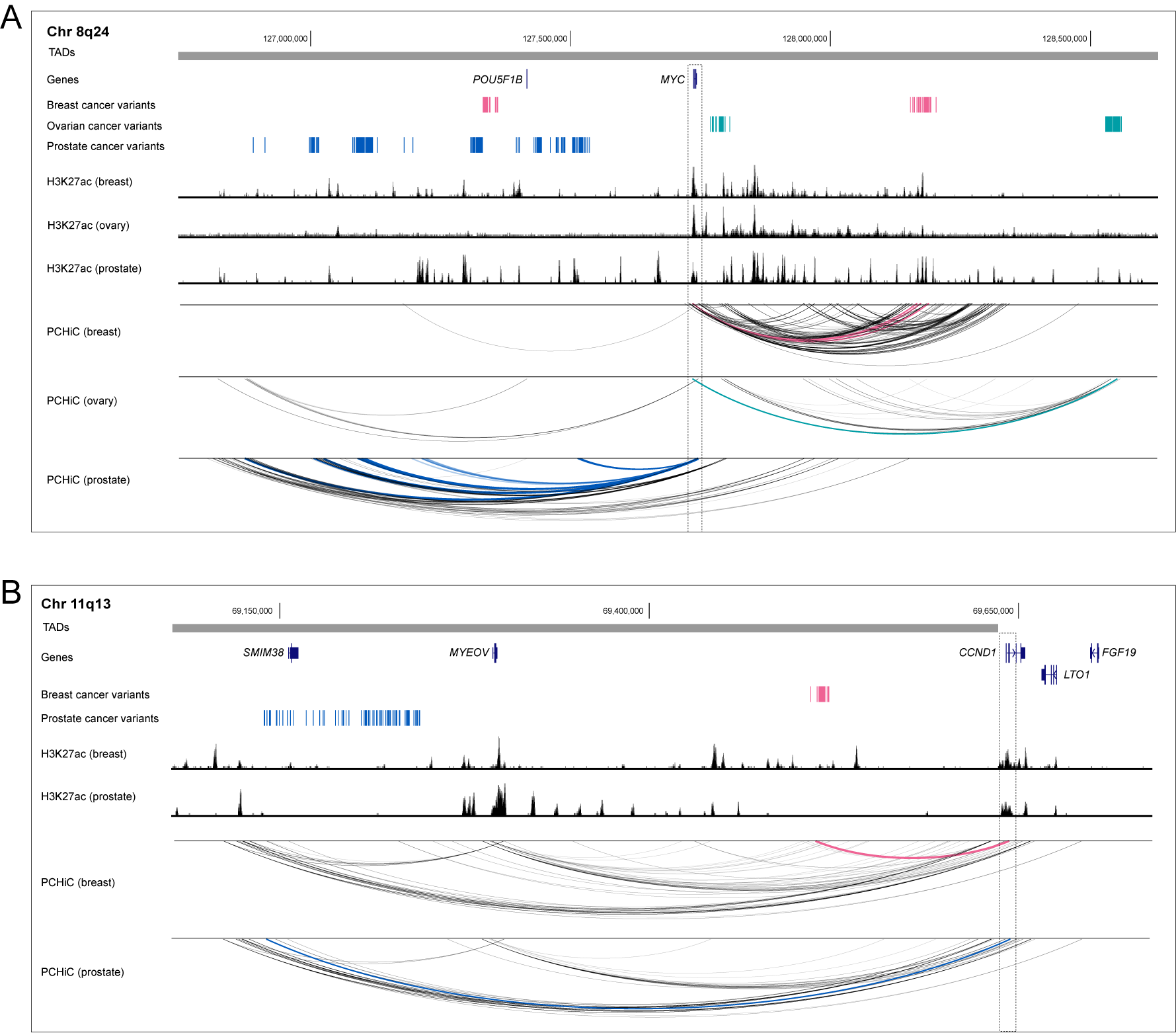

Supplement: S4 Fig — (A). WashU genome browser (hg38) showing TADs as horizontal gray bars above GENCODE genes (blue). The risk variants are shown as pink (breast cancer), teal (ovarian cancer) and blue (prostate cancer) vertical lines. The H3K27ac tracks from breast, ovary and prostate cells are shown as black histograms. CHiCAGO-scored interactions are shown as colored arcs. The dashed gray outline highlights the target gene (MYC). (B). WashU genome browser (hg38) showing TADs as horizontal gray bars above GENCODE genes (blue). The risk variants are shown as pink (breast cancer) and blue (prostate cancer) vertical lines. The H3K27ac tracks from breast and prostate cells are shown as black histograms. CHiCAGO-scored interactions are shown as colored arcs. The dashed gray outline highlights the target gene (CCND1). (TIF) [file pgen.1011490.s004.tif]

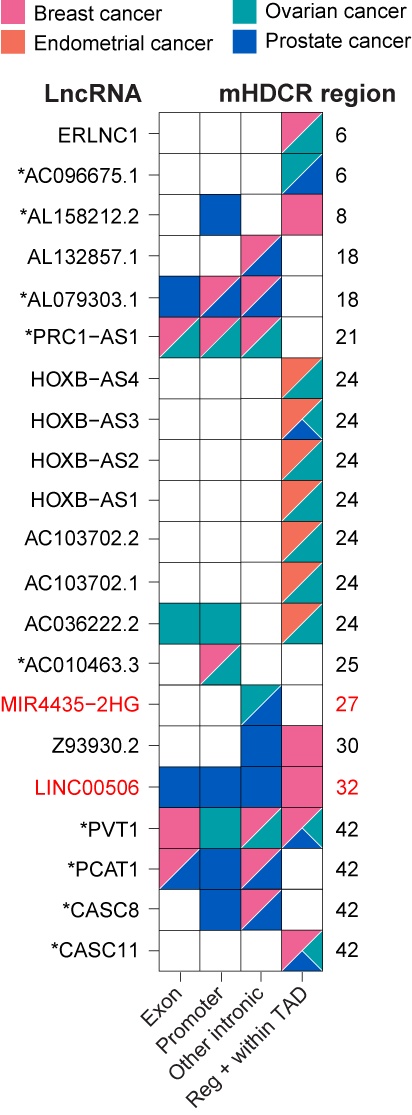

Supplement: S5 Fig — Summary of candidate mHDCR lncRNAs that are associated with two or more HDCs (pink-breast cancer; orange-endometrial cancer; teal-ovarian cancer; blue-prostate cancer). The left y-axis shows the lncRNA names, the right y-axis denotes the mHDCR region. The asterisk denotes genes with evidence of cell-type-specific regulatory activity from ChromHMM. The red text highlights lncRNAs that are the only identified target at the mHDCR region. The x-axis provides the location and/or functional annotation of the HDC risk variants. Exon-variants located in lncRNA exons; Promoter-variants located in lncRNA promoters defined as TSS ± 2 kb; Other intronic-variants located in lncRNA introns and RegulomeDB scores indicate the variants are regulatory; Reg + within TAD-variants located outside any lncRNA transcripts, RegulomeDB scores indicate the variants are regulatory, the PCHiC data show chromatin interactions between the variant and lncRNA promoters and the interactions are within defined topologically associated domain (TAD) boundaries. (TIF) [file pgen.1011490.s005.tif]
