# Supplementary material for: GWAS and 3D chromatin mapping identifies multicancer risk genes associated with hormone-dependent cancers
Source: PLoS Genet. 2024 Nov 25;20(11):e1011490. doi: 10.1371/journal.pgen.1011490 (PMC11627375; doi:10.1371/journal.pgen.1011490)
Supplement: Attachment — Submitted filename: Response to Reviewers.pdf [file pgen.1011490.s015.pdf]

<u>REVIEWER 1</u>: In this paper, Rivera et al described GWAS together with 3D genome organization mapping in order to identify multicancer risk genes that are associated with hormone-dependent cancers such as prostate cancer, breast cancer, etc. This is a useful study, as it helps scientists identify multicancer risk genes that could be studied further for the development of new drugs, and so on. Overall, I found the study clear and easy to understand. I also appreciated the detailed Supplementary Tables with lists of targets. I recommend this study for acceptance in PLoS Genetics and do not have any further suggestions or comments.

We thank the reviewer for their supportive comments.

<u>REVIEWER 2</u>: It has been proposed that hormone-dependent cancers (HDCs) have a degree of common genetic etiology. The authors of this manuscript sought to determine the degree of such sharing and then implicate causal variants and genes at those loci shared across at least two traits. This is done with the integration of public domain and lab-generated datasets. Although the results represent an advance in the field, there are some concerns:

1. The authors state that "most HDC risk variants are noncoding" - it would be helpful for the reader to know this number within the main text.

We have now added the following text "In total, 134 (~6%) of the risk variants mapped to protein-coding gene exons, while 4132 (~94%) were located in noncoding regions (intergenic, intronic and promoter regions)." (Page 5; lines 103-105). We have also added the number of exonic, promoter, intronic and intergenic variants for each cancer type to Fig S1A.

2. The authors then go on to say "the promoter and intronic variants were further explored for regulatory functions using RegulomeDB" - there needs to better description in this context; in particular, were the cell types driving these observations relevant to these traits? If not, then this would be of concern. And it doesn't appear public domain or in-house generated ATAC-seq datasets were leveraged, which would give unbiased insights into where potential regulatory regions exist in the given cell types based on open chromatin.

We have now included more detail about the RegulomeDB analysis. "Pre-calculated RegulomeDB scores for dbSNP v153 common SNPs (MAF ≥ 0.01) were downloaded from regulomedb.org. From this dataset, we extracted two key metrics: the RegulomeDB ranking, where a score of 1 indicates stronger supporting evidence for regulatory function and probability scores, where a score of 1 indicates higher likelihood that a variant is regulatory." (Page 5; lines 109-113)

We have also analysed the overlap of putative functional variants with ChromHMM data from EpiMap (20). Data were available for three breast, five prostate, three ovarian and one endometrial tissue sample or cell line. We annotated all potential regulatory variants using cell type-specific chromHMM signatures and have now included this data in **Tables S4, S5** and **S9**.

Based on this additional data, we have made the following additions to the text. "ChromHMM data was available for three breast, five prostate, three ovarian and one endometrial tissue sample or cell line. We annotated the chromatin states of all potential regulatory variants using ChromHMM signatures (20) (**Table S4**)." (Page 5; lines 113-116)

“For those candidate mHDCR genes with promoter variants, all were supported by evidence of cell-type-specific regulatory activity from ChromHMM (**Table S4**).” (Page 5; lines 125-126)

“Twenty-five genes had evidence of cell-type-specific regulatory activity based on ChromHMM data from the corresponding cell types (**Fig 4A** and **Table S5**), and 19 were supported by evidence from a single cancer type (**Table S5**). The remaining genes were retained as mHDCR candidates, as their regulatory activity may be context-dependent or associated with specific cell or cancer subtypes.” (Page 7, lines 164-168).

3. The authors refer to their chromatin conformation capture approach as high resolution. This reviewer would argue that the use of HindIII, a 6-cutter, is actually low resolution by today’s standards given there are now even commercially available options based on 4-cutter resolution. Indeed, at this low resolution used, there is concern that there is a lack of precision in the genes implicated.

We have now removed ‘high resolution’ from the manuscript and have acknowledged this limitation in the discussion. “We also acknowledge that using a 6-base pair (bp) cutter in PCHiC reduces resolution and introduces specific biases compared to more recent methods employing 4-bp cutters.” (Page 11, lines 214-216).

4. The investigators use "breast, endometrial, ovarian and prostate non-tumorigenic and cancer cell lines". Given the approach is to understand susceptibility variants to the given diseases, it would seem more relevant to assess their interaction landscape before disease onset. The concern would be that looking in post-onset settings there is dysregulation in the interactions and thus assessment of the priming of the susceptibility is clouded.

We intentionally selected multiple non-tumorigenic and cancer cell lines for each HDC type. A common misconception that GWAS variants are solely associated with disease initiation. However, our research (Michialidou et al, Nature 2017), along with other studies, have shown that GWAS variants are enriched in cancer cell lines, with a significant proportion overlapping regulatory sites, suggesting many of the variants may play a role in cancer development.

REVIEWER 3: The manuscript by Rivera et al explores a descriptive pipeline to identify genes associated with multiple hormone dependent cancers (HDCs). They use genomic data to identify genes associated with genetic loci that are related to multiple HDCs and show that these are enriched in cancer genes as well as known drug targets. The manuscript is very clearly written, and the results are demonstrated very well. I have some further points that might be good to address:

1. When defining mHDC regions, the authors are merging two HDC variants if they are within a certain neighborhood. But if they are still independent/in low LD, should they be not considered as separate HDC regions rather than merging into one mHDC regions? The downstream target gene analysis might still be valid if two low-LD variants, corresponding to different HDCs, have independent effects on the same gene.

We did not take LD between traits into account when defining our mHDC regions. We considered the set of variants for each trait based on genomic location rather than LD, then used functional genomic data to identify common target genes. To further clarify we have included an additional

sentence in the text. “We did not consider linkage disequilibrium (LD) between variants of each cancer type, focussing solely on their positional relationships.” (Page 4, lines 87-89).

2. I am curious as to what led the authors to favor investigating promoters as opposed to others like enhancers for which high quality variant-gene mappings are available? In general, there are a plethora of methods & scores that assign variants to genes based on their putative regulatory activity (e.g. work led by Alkes Price’s group and others), which possibly incorporated HiC data as well.

Numerous informatic methods are available that link regulatory variants to target genes, often using eQTLs or TWAS, each with its own strengths and limitations. Our approach involved a gene-centric analysis that remained agnostic to promoters and enhancers in relevant HDC cell types. Much of the PChiC data generated in the study is not yet available for the relevant cell types or included in current analytical pipelines. We believe this study offers a complementary approach that may prove useful for future functional studies.

3. How was the overlap analysis for drug targets done? Why was one drug repository chosen? If drug repurposing is one of the final goals, I would imagine, looking at multiple drug databases would give a more robust result.

We have now included more details of the Citeline drug target overlap analysis in the text, as follows: “Pharmacological target data were sourced from a recent study (36). To evaluate the significance of overlaps between known drug targets and candidate mHDCR genes, we applied Fisher's exact test, using a background set consisting of all candidate genes across all mHDCRs. For visualization of the enrichment, we compared the number of known drug targets to randomly sampled gene sets, matched to the number of genes per mHDCR region, performing 10,000 iterations and z-scaling the background distribution. A similar approach was used to assess the overlap between mHDCR genes and genes from the COSMIC.” (Page 8, lines 192-199).

We chose the Citeline Pharmaprojects database because it offers the most up-to-date information on compounds that have progressed through clinical trials. Other databases do offer similar information, but performing the same analysis across multiple platforms is challenging and complicates achieving statistical significance.

4. Given that the authors are looking for multi-HDC targets, analysis of GWAS with striking disparate power/sample size will skew the results towards discovering genes with effect on breast and/or prostate cancers.

This is true. We noted this sample size disparity in the main text (Page 4, lines 93-95), but have now also addressed it as a limitation for target gene discovery in the discussion section. “The majority of candidate mHDCR genes (45/53) were associated with breast and/or prostate cancers, which is likely attributable to the larger GWAS sample sizes for these cancers.” (Page 7, lines 158-159).

5. Since identifying gene targets for multiple cancers is the theme, I would expect that the genes are enriched in hormone dependent processes and/or immunological processes. Can the authors investigate that?

Due to the limited number of candidate target genes, conducting comprehensive pathway analyses is challenging. We have attempted a targeted approach, using the genes in the mHDCR regions as

the background set (N = 3097), through which we identified gene ontology pathways predominantly linked to developmental processes (see below). However, given the targeted nature of this analysis, which may bias the pathway results, we decided not to include the findings in the manuscript.

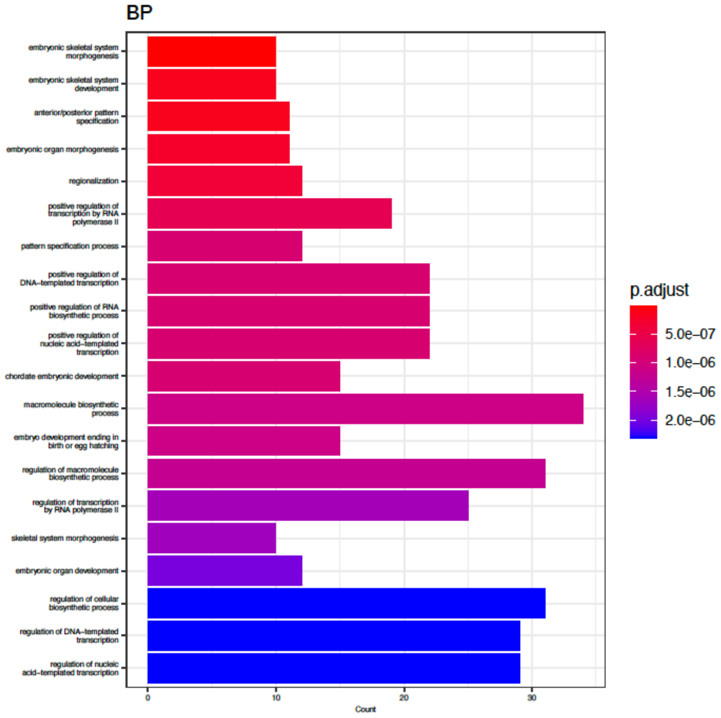

6. In my opinion, although multi-HDC genes are important, the genes associated with single HDCs are also equally important, though slightly away from the overall goal, especially for the cancers which have lower power. Can the authors incorporate this in the discussion?

Although this was not the primary focus of the study, we did identify more than 300 candidate genes linked to individual HDCs (listed in new **Table S9**). We have now included additional information in the discussion to highlight this point. "One possible explanation is that individual HDCs are driven by distinct genes. In fact, we identified over 300 genes associated with individual HDCs across 37 regions (**Table S9**). However, it is important to note that this list is not exhaustive due to the limitations of our targeted approach." (Page 9, lines 204-207).
